# The effect of nutrition education based on PRECEDE model on iron deficiency anemia among female students

**DOI:** 10.1186/s12905-021-01394-2

**Published:** 2021-06-24

**Authors:** Ali Khani Jeihooni, Sanaz Hoshyar, Pooyan Afzali Harsini, Tayebeh Rakhshani

**Affiliations:** 1 Department of Public Health, School of Health, Shiraz University of Medical Sciences, Shiraz, Iran; 2 Department of Public Health, School of Health, Fasa University of Medical Sciences, Fasa, Iran; 3 Department of Public Health, School of Health, Kermanshah University of Medical Sciences, Kermanshah, Iran

**Keywords:** Iron-deficiency anemia, Students, Nutrition, Education, PRECEDE model

## Abstract

**Background:**

Iron deficiency anemia disrupts the concentration of adolescent girls; reduces their academic achievement, productivity, and physical strength, and increases the risk of infection. This research aim was to evaluate the effectiveness of the PRECEDE model nutrition education on iron deficiency anemia among female students of Fasa City, Fars Province, Iran.

**Methods:**

This quasi-experimental study was done on 160 students (80 experimental and 80 control groups) who were selected using a random sampling method in Fasa City, Fars Province, Iran, in 2018–2019. The educational intervention included six sessions based PRECEDE model for 45 or 50 min. A scale of this study consisted of two parts; demographic information, and PRECEDE constructs were used to determine the nutritional behaviors status concluding preventing iron deficiency anemia and hemoglobin, hematocrit, and ferritin blood level in two (before and 4 months after intervention) times.

**Results:**

In the experimental group of the students the mean age was 13.85 + 1.72 years and in the controlled group was 13.60 + 1.81 years. Moreover, there was no significant difference in the PRECEDE constructs, and nutritional behaviors preventing iron deficiency anemia before the intervention in two groups of study. However, the experimental group showed a significant increase 4 months after the intervention. Also, there was no significant difference in the mean score of hemoglobin, hematocrit, and ferritin blood level between the two groups before the intervention. However, in ferritin level, a significant increase was shown in 4 months after the intervention in the experiential group.

**Conclusions:**

Based on results, the nutrition intervention education base on PRECEDE model has a positive effect to improve iron deficiency anemia preventive behaviors in female students.

## Background

Iron deficiency anemia is communal health disruptive in wholly countries, with a prevalence in developing countries approximately 3 to 4 times higher [1]. Prevalence of iron deficiency anemia (IDA) among females after puberty because of menstrual bleeding increases [2]. After marriage, they suffer from severe iron deficiency during pregnancy. In these mothers, not only the risk of death due to bleeding during labor increase but also infants born are underweight and have low iron stores [3]. Iron deficiency anemia during pregnancy and the first two years of life have irreversible effects on infants’ brain growth, which reduces their IQ [4, 5].

Iron deficiency is the most important cause of anemia [6] that about 50% of anemia is due to iron deficiency [6]. Iron deficiency anemia in pregnancy is associated with consequences such as the increased risk of maternal mortality, preterm birth, low birth weight, infants with low iron stores, cognitive decline, decreased learning, and performance of children in school [7–9]. It also disrupts the concentration of adolescent girls, decreases their educational success, productivity, and physical strength, and increases the risk of infection [7]. Iron deficiency anemia reduces the capacity of red blood cells delivering oxygen to the tissues of the body, with clinical symptoms such as conjunctival pallor, shortness of breath, dizziness, and weakness [10]. According to the World Health Organization, 25% of students have iron deficiency anemia and the prevalence of IDA among the student and adolescent population is reported to be 29.2 to 79.6% [11]. Karkar examined the prevalence of anemia among nursing students, which showed that 60% of adolescent girls had anemia [12].

In a study conducted on the staff of Vali-Asr hospital of Fasa in the first 4 months of 2016, the prevalence of iron deficiency anemia was reported to be 5.8%. Quality of life in patients with iron deficiency anemia is significantly lower than healthy individuals in terms of physical, mental, and social health [13]. Adolescence is a critical period in which important changes occur for humans. Deficiency of specific nutrients reduces energy supplies, decreases muscle strength, and causes disorders such as anemia and immune deficiency [14]. Adolescents have a limited ability to make informed choices and are dependent on external factors [15]. Many health challenges among adolescents begin with their behavioral choices, and schools have a unique opportunity to use Health education programs to influence behavioral choices [16]. Iron deficiency anemia disrupts the concentration of adolescent girls; reduces their academic success, productivity, and physical strength, and increases the risk of infection [17]. The economic and social burden of anemia has made it a major challenge in Iran [18–20]. Therefore, given the increase of anemia in the country and promoting anemia control behaviors, intervention through the implementation of behavior change patterns is essential. Also, the need to educate people about the preventive behaviors and lifestyle changes associated with anemia is strongly felt [21, 22].

Evidence shows that nutrition education reduces the prevalence of certain diseases, including anemia in many European, North American, Asian, and Australian countries. Therefore, as appropriate, nutritional recommendations and implementation of nutrition education programs seem necessary. Considering the important role of schools in education, implementing a nutrition education program in schools was suggested as an appropriate intervention to increase nutritional awareness [23].

Shahnazi et al. found that in the experimental group, the mean score of the PRECEDE constructs, and preventive behaviors of iron deficiency anemia improved significantly after intervention [24].

Shakouri et al. showed a positive effect of intervention in the case group which influenced students' knowledge and attitude about iron deficiency anemia [25]. Latifi and Dehdari showed that 0.2% of students had poor eating habits, 59% had moderate eating habits, and 40.8% had appropriate eating habits to prevent iron deficiency anemia [26].

Considering the framework of PRECEDE model, its constructs; the role of students' knowledge, attitude and self-efficacy, reinforcing factors (family and school authorities), and enabling factors (access to information and educational resources in eating behaviors preventing IDA), we used the PRECEDE model, which has been introduced as a successful model in many clinical and field trials [21, 25]. This model provides a framework by which factors affecting behavior such as predisposing factors (knowledge, attitude, etc.), reinforcing factors (family, peers, etc.), and enabling factors (access to resources, skills, etc.) in the diagnosis of education are identified [27]. The PRECEDE–PROCEED model systematically plans, implements, and evaluates health education activities. It is designed with an ecological approach in interconnected stages and a logical process [21]. PRECEDE–PROCEED consists of nine phases, including four planning phases, one implementation phase, and three evaluation phases. Its main approach is to identify the desired outcome, determine the causes of the desired outcome, and intervene to achieve the desired outcome [21].

Various studies have been conducted based on educational models in the prevention of iron deficiency anemia among students, including Abalkhail et al. [28], Abedini et al. [29], Shahnazi et al. [24], Shakouri et al. [25], Fathizadeh et al. [30], Falahi et al. [31] and Mirzaei et al. [32].

Given the importance of prevention of iron deficiency anemia, the necessity of nutrition education for girls, and the lack of study on female students of Fasa, the present study aimed to determine the effectiveness of nutrition education based on the PRECEDE model on iron deficiency anemia among female students of Fasa.

## Methods

This quasi-experimental study was performed on 160 seventh and eighth grades female students of Fasa. The sample size was determined 120, with 95% confidence and 80% power based on similar texts [30, 32]. Due to the probability of sample loss, 160 patients were included in the study and divided into two groups of 80 experiential and controlled groups.

Out of twenty-four girls' public high schools, four schools were randomly selected (two as the experiential group and two as the controlled group), with forty students from each school. Samples were to be volunteers and study at one of the public high schools.

Inclusion criteria were 7th and 8th-grade high school students, written informed consent, and not having iron deficiency anemia.

Exclusion criteria included unwillingness and absence of more than two in the sessions.

The data gathering tool was based on Eftekhar Ardebili et al. [33], Shahnazi et al. [24], and Shakouri et al. [25] study that validity and reliability of the questionnaire were confirmed.

This tool was including demographic information and a questionnaire based on the PRECEDE model. A 15-item multiple-choice questionnaire (scoring between 0 and 15) was designed to measure the knowledge. The attitude was measured by a 10-item questionnaire, based on the Likert scale ranging from ‘completely disagree’ (score 1) to ‘completely agree’ (score 5). Self-efficacy was measured by a 10-item questionnaire (scoring between 0 and 40); for example, ‘Can you take iron supplements once a week?’ The answers ranged from ‘not at all (score 0) to ‘very much (score 4). The reinforcing factors were measured by 8 items (scoring between 0 and 32). The answers based on the Likert scale ranging from ‘not at all (score 0) to ‘very much (score 4). Enabling factors were measured using 6 items (scoring between 0 and 24); for example, ‘To what extent does one have access to iron-rich foods?’ The answers based on the Likert scale ranging from ‘absolutely no’ (score 0) to ‘completely yes’ (score 0). Nutritional behaviors to prevent iron deficiency anemia were measured by 10 questions (scoring between 0 and 20). The answers were ‘yes’ (score 2), ‘somewhat’ (score 1), and ‘no’ (score 0).

The item effect size higher than 0.5 and content validity ratio more than 0.79 were considered to evaluate the validity of the questionnaire. Also, the face validity, a list of items was checked by middle school female students. The content validity was consulted by 12 specialists and professionals in health education and promotion (*n* = 10) and nutritionists (*n* = 2) (outside the research team). based on Lawshe’s table, items with CVR > 0.56 for 12 specialists were considered acceptable; Which, for most of the item values were > 0.70. The whole reliability of the scales was 0.89 using Cronbach's alpha. Reliability of construct of the model was the knowledge: 0.84, attitude: 0.88, self-efficacy: 0.82, reinforcing factors: 0.79, enabling factors: 0.85, and nutritional behaviors: 0.90 preventing iron deficiency anemia respectively.

The questionnaire was completed by two groups before the intervention.

With the written consent of the parents and coordination with the school authorities, 2 ccs of fasting blood (1 cc for the Ferritin test and 1 cc for the CBC test) were drawn from each student. The blood samples were transferred to the laboratory immediately and all experiments were performed in a laboratory with pre-set devices.

The intervention for the experimental group consisted of materials such as small group discussions, Q&A, practical demonstration, videos, PowerPoint, and booklet in 45–50 min of six sessions.

Participants of the experimental group were divided into groups with 8 members for reinforcing factors construct (friend group-small group) and the educational program was performed for 10 groups with 8 members (80 subjects of experimental group).

The program was administered by health education—promotion and nutrition professionals, also, two nutrition and adolescent health experts from the Center of Health in Fasa were of assistance. In these sessions, the importance of nutrition, the prevalence of anemia and its risk factors, and nutritional behaviors preventing iron deficiency anemia were discussed. A session was held once a week with teachers, school officials, a member of the family, and staff of centers of health as subjective norms and social supporters.

## Summary of education sessions

- Session 1: Understanding the role of red blood cells and iron in the body, the symptoms of iron deficiency anemia and its prevalence in adolescent girls, the reasons for the need for iron in girls, and different types of iron in food
- Session 2: Understanding the prevalence of iron deficiency anemia in adolescents, predisposing factors of iron deficiency and its consequences
- Session 3: Good diet, the importance of a good diet, the impact of iron-rich diet on relieving fatigue, happy life, and introducing the students to iron tablets
- Session 4: The reinforcing role of supporters in the provision of proper nutrition, proper cooking, low-cost iron-rich food substitution for high-cost iron-rich foods, solutions to eliminate wrong habits of taking iron pills in schools, forming a WhatsApp group to exchange information, and holding the session with parents, school officials, and health care, staff
- Session 5: Accessing the resources and searching nutritional information, the importance of student diet, lecture by a patient with iron deficiency anemia on the complications and burden of the diseases
- Session 6: Reviewing past sessions, providing a booklet, dividing participants into 10-men groups, nutritional behaviors

To follow the activities, an education session, for the students was thought almost once a month, and a WhatsApp group was formed to exchange information for the parents, with at least five educational and encouraging messages sent to the parents per week. For ethical concerns, at the end of the study for the control group was held an education session. Four months after the intervention, blood sampling and questionnaires were completed. Analyzing data was done by SPSS software through paired or independent t-test, and χ^2^ test, and a significant level was considered *p* < 0.05.

## Findings

In the present study, 160 7th and 8th-grade high school female students participated. The mean age of the students was 13.85 + 1.72 years in the experiential group and 13.60 + 1.81 years in the controlled group, respectively. There wasn’t any significant difference between the two groups based on the independent t-test (*p* = 0.204). Regarding the education level (*p* = 0.144), household monthly income (*p* = 0.202), father’s education (*p* = 0.198), mother’s education (*p* = 0.186), mother’s occupation (0.331), fathers' occupation (*p* = 0.221), there was no statistically significant difference between the two groups (Table 1). The results also showed that there was no significant difference between the two groups in terms of knowledge, attitude, self-efficacy, reinforcing factors, enabling factors, and nutritional behaviors preventing iron deficiency anemia before the intervention; however, after the intervention, the experimental group presented a significant increase in the 4 months later (Table 2).

**Table 1:** Comparison of the frequency distribution of demographic variables in the experimental and control groups

Variables	Experimental group		Control group		*p* value
	Number	Percentage	Number	Percentage	
*Educational stage*					
7th grade	46	57.50	48	60	0.145
8th grade	34	42.50	32	40	
*Household monthly income*					
Less than 2 million tomans	26	‏32.50	22	27.50	0.210
2–5 million toman	34	42.50	35	43.75	
More than 5 million tomans	20	25	23	28.75	
*Mother’s occupation*					
Employed	22	27.70	19	23.75	0.331
Housewife	58	72.50	61	76.25	
*Father’s occupation*					
Employed	22	27.50	24	30	0.212
Self-employed	28	35	26	32.50	
Laboror	17	21.25	15	18.75	
Unemployed	13	16.25	15	18.75	
*Mother’s education*					
Illiterate	3	3.75	2	2.50	0.186
Primary school	14	17.50	18	22.50	
Secondary school	17	21.25	16	20	
High school	36	45	32	40	
College	10	12.50	12	15	
*Father’s education*					
Illiterate	2	2.50	1	1.25	0.198
Primary school	12	15	10	12.50	
Secondary school	18	22.50	24	30	
High school	34	42.50	30	37.50	
College	14	17.50	15	18.75

**Table 2:** Comparison of mean score of PRECEDE model constructs in the experimental and control groups before and after the intervention

Variable	Group	Before intervention M ± SD	4 months after the intervention M ± SD	*p* value
Knowledge	Experimental	7.34 ± 1.26	12.98 ± 1.35	< 0.001
	Control	7.78 ± 1.81	8.14 ± 1.70	0.327
	*p* value	0.164	*p* < 0.001	
Attitude	Experimental	20.34 ± 4.11	42.25 ± 4.55	< 0.001
	Control	21.62 ± 4.08	22.53 ± 4.26	0.258
	*p* value	0.173	*p* < 0.001	
Self-efficacy	Experimental	11.32 ± 3.62	33.39 ± 3.74	< 0.001
	Control	11.96 ± 3.87	12.70 ± 3.89	0.147
	*p* value	0.347	*p* < 0.001	
Reinforcing factors	Experimental	14.40 ± 2.65	28.11 ± 2.09	< 0.001
	Control	14.12 ± 2.80	15.22 ± 2.46	0.169
	*p* value	0.228	*p* < 0.001	
Enabling factors	Experimental	9.42 ± 1.16	20.35 ± 1.29	< 0.001
	Control	8.84 ± 1.72	9.16 ± 1.79	0.164
	*p* value	0.256	*p* < 0.001	
Preventive behaviors	Experimental	6.51 ± 1.25	17.65 ± 1.09	< 0.001
	Control	6.86 ± 1.12	7.50 ± 1.18	0.192
	*p* value	0.317	*p* < 0.001	

Based on results, before the intervention between the two groups, there was no significant difference in the mean score of hemoglobin, hematocrit, and ferritin blood. However, 4 months after the intervention, a significant increase in ferritin level showed in the experimental group (Table 3).

**Table 3:** Comparison of mean score of hemoglobin, hematocrit, and ferritin in blood samples of the experimental and control groups before and after the intervention

Variable	Group	Before intervention M ± SD	4 months after the intervention M ± SD	*p* value
Hemoglobin	Experimental	12.34 ± 0.86	12.56 ± 0.85	< 0.065
	Control	12.58 ± 0.82	12.46 ± 0.79	0.188
	*p* value	0.084	*p* < 0.069	
Hematocrit	Experimental	35.76 ± 2.53	35.65 ± 2.55	< 0.154
	Control	35.22 ± 2.58	34.84 ± 2.50	0.162
	*p* value	0.093	*p* < 0.084	
Ferritin	Experimental	30.32 ± 18.62	37.29 ± 19.44	< 0.001
	Control	31.26 ± 19.38	30.73 ± 19.80	0.159
	*p* value	0.425	*p* < 0.001	

## Discussion

Given the increase of anemia in the country and promoting anemia control behaviors, intervention through the implementation of behavior change patterns is essential. Also, the need to educate people about the preventive behaviors and lifestyle changes associated with anemia is strongly felt [24, 30]. The present study was aimed to evaluate the effectiveness of the model-based of nutrition education on iron deficiency anemia among the 7th and 8th-grade high school female students of Fasa. In the present study, there wasn’t any significant difference between groups of study in demographic status, mean score of PRECEDE constructs, preventive behaviors of iron deficiency anemia, hemoglobin, hematocrit, and ferritin before the educational intervention. The results also showed that the mean score of knowledge and attitude increased significantly in the experiential group 4 months after the intervention, indicating the influence of the PRECEDE model on increasing knowledge and positive attitudes toward eating behaviors preventing anemia. Education sessions through videos, small group discussions, booklets, and a lecture by a patient with iron deficiency anemia for the experimental group increased the students' knowledge and positive attitude toward eating behaviors preventing anemia.

In a quasi-experimental study by Mansourian et al., nutrition education increased the knowledge, attitude, and performance of the experimental regarding eating behaviors preventing IDA 6 weeks after the intervention [34]. Vaezi et al. investigated the impact of a multimedia educational package preventing iron deficiency anemia on the health literacy of female students. The results showed that the mean score of knowledge, attitude, and performance of students in the experimental group immediately and 1 month after the intervention showed a significant increase. The most important sources of information about iron deficiency anemia were teachers, medical and health teams, and friends [35]. In the studies of Otoo, Robertson, Abd Elhameed, and Ayub et al. [36–39], the educational intervention increased women's knowledge of iron deficiency anemia. Also, in the studies by Heidarnia, Hazavehei, and Mehrabian et al. [40–42], the educational intervention increased the knowledge and attitude of patients toward anemia, which was consistent with the results of the present study.

The educational intervention increased the perceived self-efficacy of the experimental group. The perceived self-efficacy is a powerful source of motivation and, in fact, judging people’s organizing ability to achieve certain goals. People with high perceived self-efficacy are more determined during times of challenge and spend more time and effort. Such individuals, even after failure, are more likely to maintain healthy behaviors and have a stronger motivation to set and achieve goals [43].

In an educational intervention study by Ghoreishi et al., which was two 90-min sessions for the experimental group; the educational intervention increased the mean score of self-efficacy, knowledge, and iron supplementation behavior in the experimental group [44]. In the study of Roshan et al., self-efficacy improved in the experiential group after the educational intervention [45].

In Ghaderi et al. study, the educational intervention increased knowledge, self-efficacy, and preventive behaviors of anemia [46]. In the study of Mohammadzadeh Larijani et al., Self-efficacy predicted behaviors preventing iron deficiency anemia [47]. In a quasi-experimental study by Mirzaei et al., the mean score of self-efficacy and students’ performance regarding preventive behaviors increased 3 months after the intervention [32]. In line with the enabling factors showed a significant difference between the experiential and controlled groups after the intervention. Also, education sessions through videos, small group discussions, booklets, and a lecture by a patient with iron deficiency anemia for the experimental group increased the students' knowledge and positive attitude toward eating behaviors preventing anemia.

In a study by Ardebili et al., the mean score of enabling factors in the experimental group showed a significant increase in 3 months after the intervention [33]. In Shahnazi et al. study, the intervention for the experimental group which, consisted of three 60-min educational sessions, the mean score of enabling factors and preventive behaviors of iron deficiency anemia increased significantly in the experimental group in 3 months after the intervention [24]. The results of other studies were along with the results of the current study [30, 48–51].

The results showed that there was a significant difference between the two study groups regarding the reinforcing factors 4 months after the educational intervention, indicating the positive impact of the reinforcing factors based on PRECEDE model. Education sessions for parents, teachers and school officials, and staff of health care as subjective norms and social supporters through group discussions and forming a WhatsApp group for parents improved the mean score of reinforcing factors in the experimental group. In the study of Rai et al., social support was a determinant of iron and folic acid supplementation in pregnant women [52].

In a study by Zarei et al., the educational intervention increased the mean score of enabling factors, predisposing factors, and iron intake from 68 to 95% 3 months after the intervention [53]. In a study by Mehrabian et al., the mean score of subjective norms and enabling factors in the experimental group increased significantly three and a half months after the intervention [42]. In a quasi-experimental study by Jalambadani et al., the mean score of knowledge, attitude, perceived behavioral control, intention, and use of iron tablets were significantly increased in the experimental group 3 months after the intervention [54]. The results of the other studies were consistent with the results of the present study [55–57].

The present study showed that in the experimental group, the mean score of dietary behaviors preventing iron deficiency anemia showed a significant increase 4 months after the educational intervention, while in the other group of participants there was no significant variation. The results also showed that the level of ferritin in the experimental group was significantly higher than that of the control group 4 months after the intervention. There was no significant increase in hemoglobin and hematocrit levels after the intervention. An increase in behavior score and ferritin level in the experimental group indicated the effect of knowledge, attitude, self-efficacy, reinforcing factors, and enabling factors on promoting nutritional behaviors to prevent iron deficiency anemia. In a study by Sharifi Rad et al., the mean score of PRECEDE model constructs and preventive behaviors were significantly increased in the experimental group 3 months after the intervention. However, there was no significant difference between the hemoglobin and hematocrit of the two groups [58]. In a quasi-experimental study by Falahi et al., hemoglobin, ferritin, and zinc increased after the intervention [31]. In a study by Amer AL-tell et al., dietary behaviors and hemoglobin levels increased in the experimental group [59]. In the study of Mehrabian et al., the BASNEF model educational intervention improved the nutritional behavior preventing iron deficiency anemia and increased ferritin level in the experiential group [42].

In a study by Jalambadani et al., the theory of planned behavior educational intervention improved iron supplementation behavior and increased ferritin level [54]. In a study by ALaofe et al., the mean score of knowledge, iron intake, hemoglobin, and Ferritin showed a significant rise after the intervention in the experimental group [60]. A study by Araban et al. also showed that nutritional education based on Health Belief Model increased iron and folic acid intake in pregnant women [61]. The results of the present study were consistent with the results of the other studies [30, 44, 62–66].

## Conclusion

The results of the present study showed that the PRECEDE model could increase the ferritin level in adolescent girls by improving the mean score of knowledge, attitude, self-efficacy, reinforcing factors, enabling factors, and nutritional behaviors. This reduces iron deficiency anemia and its consequences and prevention of the grave problem with anemia during pregnancy. Therefore, to improve the health of adolescent girls, especially in the prevention of iron deficiency anemia, educational programs should be implemented using appropriate models of health education along with the active participation of girls and mothers in the education process in schools.

## Data Availability

The datasets used and/or analyzed during the current study are available from the corresponding author upon reasonable request.

## Abbreviation

IDA: Iron deficiency anemia
